# Admitting to bullying others or denying it: Differences in children’s psychosocial adjustment and implications for intervention

**DOI:** 10.1177/01650254241242690

**Published:** 2024-04-02

**Authors:** Claire F. Garandeau, Tiina Turunen, Jessica Trach, Christina Salmivalli

**Affiliations:** University of Turku, Finland

**Keywords:** Bullying, anti-bullying intervention, anti-bullying attitudes, victimization, peer status, empathy, internalizing problems, self-esteem

## Abstract

This study examined whether, for bullying perpetrators, admitting to their behavior was associated with specific psychosocial characteristics, and whether it predicted decreases in bullying behavior and a higher responsiveness to a successful anti-bullying program after 9 months of implementation. It also investigated whether participation in an anti-bullying program deterred admitting to the behavior. At pretest, our sample included 5,908 children and early adolescents (*M*_age_: 11.2 years) in 39 intervention and 38 control schools; among them, 1,304 were peer-identified bullying perpetrators (scoring higher or equal to 0.5 *SD* above the same-sex classroom mean). Regression analyses indicated that peer-identified bullying perpetrators who admitted to their behavior were more likely to suffer from internalizing problems and reported lower anti-bullying attitudes than those who did not admit to bullying others. There was no significant main effect of admitting to bullying on changes in peer-reported bullying 1 year later. However, in control schools only, those who admitted to bullying at pretest were more likely to continue bullying a year later than those who denied it. There was no evidence that participating in the anti-bullying program made it less likely for peer-identified bullying perpetrators to admit to their behavior.

In late childhood and early adolescence, the importance of peers for children’s adjustment increases. The desire to be popular among peers becomes prioritized (LaFontana & Cillessen, 2010) and leads some youth to engage in bullying behavior to achieve that goal. Despite a rise in the implementation of anti-bullying measures, especially in Nordic countries, school bullying—defined as repeated aggression against a peer in a more vulnerable position—remains prevalent in schools worldwide. In Finland, in which the current study is based, 18% of adolescents reported in 2018 being subjected to at least one type of bullying act a few times a month (Organisation for Economic Co-operation and Development [OECD], 2018). Although the central objective of any anti-bullying intervention is to make the perpetrators stop their behavior, precise knowledge of the psychological and behavioral factors related to their decision to pursue or cease their behavior is still lacking. For behavioral change to take place, individuals must first recognize that they engage in a problematic behavior (see transtheoretical model of behavior change; Prochaska et al., 1998). Surprisingly, concerning perpetrators of school bullying, the correlates and implications for intervention of admitting to their behavior (or denying it) have received little attention.

To assess bullying behavior, researchers mainly use peer-reports and self-reports. While neither methodology is without flaws, peer-reports, which identify those who have a reputation for bullying others, are considered to be more valid as they are collected via peer nominations (i.e., all participants in a classroom or grade level check the names of classmates who bully others on a class roster) and therefore rely on a larger number of informants who see their peers on a daily basis (Bouman et al., 2012). Peer-reported bullying has also been found to be more strongly correlated than self-reported bullying with objective indices such as discipline referrals from school records (Branson & Cornell, 2009). Self-reports, on the contrary, rely on a single informant and may be influenced by socially desirable responding, especially for forbidden behaviors such as bullying. For these reasons, peer-reported bullying will be used in this study as an indicator of bullying behavior.

The correlation between peer-reports and self-reports of bullying ranges from .10 to .50 across studies (e.g., Bouman et al., 2012)—suggesting a discrepancy between the level of bullying behavior that children report and the extent to which others perceive them as bullies. This limited overlap between the two measures is often rightly considered as a methodological challenge (e.g., Casper et al., 2015), but could also reveal information on whether admitting to (or denying) one’s bullying behavior matters for perpetrators’ adjustment and capacity to change. Reluctance to admit to their behavior is assumed to be prevalent among bullies (Cole et al., 2006; Pellegrini & Bartini, 2000; Tatum & Grund, 2019), and a relevant factor for intervention. For example, the possibility that bullies may deny their behavior when confronted about it motivated the development of no-blame approaches to tackling cases of bullying (Sullivan et al., 2005), as an absence of accusation deprives bullies of the opportunity to deny having bullied and allows the discussion to unfold more easily. Thus, denying or admitting to bullying might be an important factor to consider for anti-bullying work but the literature on this question is too scarce to inform practice in any meaningful way.

To fill this gap, this study uses a large sample of children and early adolescents from schools participating in the evaluation of the Finnish anti-bullying program KiVa (Salmivalli et al., 2013) to investigate four questions: (1) How prevalent is admitting to the behavior among students who do engage in the behavior, that is, to what extent do students nominated by classmates as engaging in bullying (i.e., those scoring relatively high on peer-reported bullying) report bullying others? (2) For peer-reported bullies, is admitting to their behavior associated with specific psychosocial characteristics, including internalizing problems, peer status, and moral attitudes? In other words, do “admitters” and “deniers” differ in their psychosocial adjustment? (3) Does admitting to bullying others (vs. denying it) predict decreases over time in the bullying behavior of peer-identified bullies and their greater responsiveness to the KiVa anti-bullying intervention? And, (4) does participating in the KiVa program deter the admission of bullying behavior? All analyses will be conducted with a subsample of peer-identified bullying perpetrators, as the distinction between admitting to the behavior and denying was only relevant when there was some indication that the child did engage in bullying.

## Psychosocial Characteristics Associated With Admitting to (Vs. Denying) Bullying

Two separate bodies of literature provide indication on whether admitting to one’s bullying behavior might be associated with specific psychosocial characteristics. First, studies that have examined peer- and self-reported school bullying suggest that their mental health, victimization, and peer status correlates differ. Regarding anxiety and depression, the general pattern emerging from the literature is that positive correlations are likely with self-reported bullying (e.g., Azevedo Da Silva et al., 2020; Bouman et al., 2012; Kaltiala-Heino et al., 2000; Klomek et al., 2007; Roland, 2002; Salmon et al., 1998; Seals & Young, 2003), whereas the association between peer-reported bullying and internalizing problems is often found to be not significant (Bouman et al., 2012; Juvonen et al., 2003; Vaillancourt et al., 2003). With regard to self-esteem, a meta-analysis also suggested a stronger negative relationship with self-reported compared with peer-reported bullying (Tsaousis, 2016; see also Bouman et al., 2012 and Cole et al., 2006). The association between being victimized and bullying also depends on the informant for bullying, with positive associations being more likely with self-reported bullying (Bouman et al., 2012). Research on bully-victims also shows that their prevalence tends to be higher when self-report measures are used (see Yang et al., 2016), suggesting that those who admit to their bullying behavior may be more likely to be bullied themselves.

Peer status indicators also appear to have a different pattern of relations depending on the bullying informant. Students high on peer-reported bullying tend to be perceived as popular by peers but are low in likeability or having friends (Bouman et al., 2012; Caravita et al., 2009; Juvonen et al., 2003; Košir et al., 2020; Vaillancourt et al., 2003), whereas the associations between both forms of status and self-reports of bullying are generally much weaker or even nonsignificant (Bouman et al., 2012; Košir et al., 2020). We should keep in mind that common method bias may partly explain why self-reported bullying is more strongly related than peer-reported bullying to adjustment indicators typically assessed with self-reports, such as internalizing problems, and peer-reported bullying more strongly related to adjustment indicators typically assessed with peer-reports, such as peer status. Although the abovementioned studies examined associations within entire samples of children or adolescents and not among subsamples of students perceived as bullies by their peers, this pattern of findings suggests that perpetrators who admit to their bullying behavior may be more likely to suffer from internalizing problems, lower self-esteem and victimization, and less likely to be popular and disliked than those who do not.

For children who bully others, reporting that they do not could sometimes be due to the fact that they do not see their behavior as actual bullying (e.g., they might think they are only teasing others). However, bullying being socially undesirable and condemned by school rules, it is also likely to reflect socially desirable responding, defined as the tendency to respond to self-report items in a manner that makes the respondent look good rather than to respond truthfully (Holtgraves, 2004). Research on socially desirable responding can also shed light on whether denying or admitting to one’s bullying behavior may be associated with specific psychosocial characteristics. Socially desirable responding encompasses at least two underlying constructs (Paulhus, 1988): self-deceptive enhancement, which is the tendency to endorse honestly held, but exaggerated positive self-descriptions, and impression management, which is the conscious desire to inflate socially acceptable characteristics. Both have been found to be negatively associated with anxiety, depression, and neuroticism (Holden & Passey, 2010; Otter & Egan, 2007; Taylor et al., 2003) and self-deceptive enhancement has been found to be positively associated with self-esteem (e.g., Bonanno et al., 2002). However, self-enhancement is also negatively associated with indicators of interpersonal adjustment, including likeability (Bonanno et al., 2002; Paulhus, 1998). These findings suggest that denying one’s bullying behavior may be associated with better mental health but lower likeability than admitting to bullying others.

Bullying being an immoral behavior, there are reasons to expect that admitting to it or denying it may be differentially associated with indicators of moral adjustment, such as anti-bullying attitudes or empathy. However, two competing hypotheses can be formulated. On one hand, it seems logical that the deception likely involved in denying bullying would go hand-in-hand with antisocial attitudes. Accordingly, the honesty displayed by those who admit to bullying others suggests that they would score lower in such attitudes. On the other hand, the positive relationship between moral disengagement and bullying was found to be stronger for self-reported bullying than peer-reported bullying (see Killer et al., 2019 for a meta-analysis), suggesting that those who admit to bullying may also be more likely to morally justify their transgressive behavior. Consistent with this finding, another study found that self-reported bullies reported more positive attitudes toward aggression than peer-reported bullies (Cole et al., 2006). These findings could be interpreted as an indication that those who are more honest at reporting their bullying behavior may also be more likely to report pro-bullying attitudes and to morally disengage from their actions. Moreover, a negative relationship was found between self-deceptive enhancement and antisociality (Otter & Egan, 2007) and between social desirability responding and psychopathy (Egan & Angus, 2004), suggesting that those who deny bullying others may be less likely to endorse pro-bullying attitudes and morally disengage. Therefore, we will explore the association of admitting to bullying with anti-bullying attitudes and empathy with no directional hypothesis.

## Implications for Anti-Bullying Interventions

The present study also aimed to investigate whether the denying/admitting distinction matters for universal anti-bullying interventions. First, we are interested in knowing whether admitting predicts a decrease in bullying over time and a stronger response to the implementation of an effective anti-bullying program, such as the KiVa program (see Kärnä et al., 2011). The literature provides little insight into the effects of admitting to bullying others on behavioral change. If those who deny are indeed guided by self-deception, their lack of acknowledgment to themselves that they are engaging in an undesirable behavior may prevent them from starting the steps required for behavioral change. According to theories of behavior change (Prochaska et al., 1998), successful changes first require a shift from a precontemplation stage, in which individuals are unaware of their problems (also referred to as denial), to a contemplation stage, in which individuals are aware that a problem exists and are seriously thinking about overcoming it. According to these theories, those who admit to their behavior should be more likely to decrease it regardless of the intervention as well as in response to intervention.

Second, the implementation of an anti-bullying program may influence how children respond to surveys about their bullying behavior. A program such as KiVa teaches participants to recognize bullying behavior and how serious it is (Kärnä et al., 2011). It conveys the message that bullying is not acceptable. For those who continue bullying others despite being exposed to the intervention, the tendency to deny their behavior might be heightened compared with perpetrators in control schools where the seriousness of bullying and the commitment of the school to fight it may be less salient. Knowing whether the implementation of anti-bullying interventions deters admitting to bullying others is of crucial importance for evaluation research.

## The Current Study

Although many studies have documented the limited overlap between self-reports and peer-reports of bullying and their different correlates in terms of intrapersonal and interpersonal adjustment (e.g., Bouman et al., 2012), little is known on whether youth identified by peers as perpetrators of school bullying and who admit to their conduct (i.e., reporting having bullied others at least once in the past couple of months) differ from those who deny it (i.e., reporting having never bullied in the past couple of months). Whether admitting to bullying makes a difference for bullying perpetrators’ continuation of the behavior and their responsiveness to anti-bullying interventions, and whether it can be affected by the implementation of an intervention also remain unknown.

This study aims to shed light on these questions. First, it provides an estimation of how prevalent denying is among children identified as bullies by their peers (i.e., scoring at least 0.5 *SD* above their same-sex classroom mean in peer-reported bullying). We expected that peer-reported bullies would display a tendency to deny their behavior, as self-reports have been found to yield underestimations of bullying compared with peer-reports (Branson & Cornell, 2009; Cole et al., 2006). Second, it examines the concurrent associations of self-reported anxiety, depression, self-esteem, empathy, and anti-bullying attitudes, as well as peer-reported victimization, likeability, and popularity with admitting to bullying in a subsample of peer-reported bullying perpetrators. We expected that perpetrators who admitted to bullying would have poorer mental health, be perceived as less popular—but not necessarily less liked—and report lower empathy and anti-bullying attitudes than those who denied it. Regarding interventions, we expected that, among peer-identified bullies, admitting to bullying would be associated with decreases in bullying 1 year later (main effect of admitting vs. denying on changes in bullying behavior) and this effect would be stronger in intervention schools (moderating effect of intervention on the association between admitting and changes in bullying behavior). Finally, we hypothesized that belonging to a school implementing an anti-bullying program would discourage admitting to bullying others among peer-identified bullying perpetrators.

## Method

### Sample

This study used data collected during the first phase of evaluation of the KiVa anti-bullying program in Finnish primary schools (see Salmivalli et al., 2010). Stratified random sampling of schools was used to include schools from all the provinces of mainland Finland (for a detailed description, see Kärnä et al., 2011). These were both Finnish-language and Swedish-language schools, as basic education in Finland is given in both languages. Half of the participating schools were randomly assigned to the intervention condition, the others were assigned as control schools and given the opportunity to start implementing the KiVa program after 1 year of serving as control schools. The sample was demographically representative of primary schools in Finland. Most children were native Finns, with the proportion of immigrants (i.e., children born outside of Finland) being 1.5%. We used both pretest data (T1), collected at the end of the school year, in 2007, prior to the beginning of the program implementation and posttest data (T2) collected at the end of the following school year, after 9 months of program implementation.

The initial sample included 8,704 children in 77 schools (including 39 KiVa intervention schools and 38 control schools). As this study uses peer nominations collected within classrooms, we selected classrooms with at least 10 students and a participation rate of at least 40% at both time points. Our sample included 5,908 children (51.1% girls; *M*_age_ = 11.25, *SD* = 0.89) belonging to 306 classrooms (175 intervention and 131 control classrooms) from 77 primary schools (39 KiVa schools and 38 control schools). The average participation in this sample was 91% at T1 and 89% at T2. The students were from Grades 3 to 5 at T1 and Grades 4 to 6 at T2. Classroom size ranged from 10 to 35 students (*M* = 22.40, *SD* = 4.70).

### Procedure

The data were collected via online questionnaires that participants filled out in the schools’ computer labs during regular school hours. They were supervised by teachers, who had been given instructions about the data collection process. To ensure the confidentiality of the surveys, each participant was assigned an ID number and was requested to use that as an individual password to log into the surveys. At the beginning of the questionnaire, the following definition of bullying (from the Revised Olweus bully/victim questionnaire; Olweus, 1996) was read to the participants and appeared on their screens: “Bullying occurs when students repeatedly perform any of the following behaviors directed towards another: say ‘mean and hurtful things or call him or her names, purposefully ignore or exclude him or her from their group of friends, hit, kick, push, shove, or tell lies or spread false rumors.’” In addition, the imbalance of power between perpetrator and target that characterizes bullying was specified: “Friendly and playful teasing is not bullying. Nor is it bullying when two more or less equally strong students argue or fight.” Participants were informed that their answers would remain strictly confidential.

In accordance with the Declaration of Helsinki, all participating children gave their assent and their parents gave written informed consent. When the KiVa research project began, neither institutional nor national guidelines required an ethics approval for noninvasive questionnaire studies. Nevertheless, this study was conducted in accordance with the recommendations of the Ethics Board of the University of Turku, Finland.

### Measures

#### Peer-Reported Bullying

Three items from the Participant Role Questionnaire by Salmivalli et al. (1996) were used to measure peer-reported bullying. Participants were asked to nominate classmates for the following behaviors: (1) starts bullying, (2) makes the others join in the bullying, and (3) always finds new ways of harassing the victim. Proportion scores were calculated for each item, by dividing the number of received nominations by the number of nominators. The three items were averaged to create a composite variable of peer-reported bullying (α = .91 at T1; α = .94 at T2).

#### Self-Reported Bullying

The global item from the Revised Olweus Bully/Victim Questionnaire (Olweus, 1996) was used for self-reported bullying. Participants were asked about their general bullying behavior: “Have you bullied another student at school during the last few months?” The items were rated on a 5-point scale: 0 = *not at all*, 1 = *once or twice*, 2 = *2–3 times a month*, 3 = *once a week*, and 4 = *several times a week*. To capture the difference between admitting to bullying others and denying it, this variable was recoded into a binary variable as follows: 0 = “not at all,” 1 = “at least once or twice.”

#### Peer-Reported Victimization

As for peer-reported bullying, victimization was assessed with three peer-nomination items from the Participant Role Questionnaire by Salmivalli et al. (1996): “He/She gets shoved and hit,” “He/She is called names and made fun of,” and “Rumors are spread about him or her.” Proportion scores were calculated for each item, by dividing the number of received nominations by the number of nominators. The three items were averaged to create a composite variable of victimization (α = .83 at T1; α = .78 at T2).

#### Psychological Adjustment

Three self-reported measures of psychological adjustment were considered: depression, anxiety, and self-esteem. Depression was measured by averaging seven items derived from the Beck Depression Inventory (BDI; Beck et al. 1996). For example, “How was your mood during the last 2 weeks?” or “How satisfied are you with yourself?” The items were rated on a 5-point Likert-type scale and formed an internally consistent scale (α = .86 at T1; α = .90 at T2). Anxiety was measured by averaging nine items (α = .88 at T1; α = .93 at T2) reflecting fear for negative evaluation and social avoidance and distress, such as “I’m afraid the others won’t like me.” Responses were given on a 5-point scale from 0 = *not at all* to 4 = *all the time*. Self-esteem was measured with 10 questions adapted from the Rosenberg Self-Esteem Scale (Rosenberg, 1965; α = .81 at T1; α = .84 at T2). The questions were slightly modified to reflect self-esteem among peers by adding the instruction to “report the way you feel about yourself when around peers.” For instance, “When I am with them, I have positive thoughts of myself.” Responses were given on a 5-point scale from 0 = *not at all* to 4 = *all the time* and averaged to create a self-esteem score.

#### Empathy for the Victim

A seven-item self-report questionnaire, designed for the evaluation of the KiVa program, was used to assess empathy toward the victim (see also Kärnä et al., 2011). Four items assessed affective empathy (i.e., the degree to which participants share the feelings of the victim), such as “When the victimized child starts to cry, I also feel bad.” Three items assessed cognitive empathy (i.e., the degree to which participants understand the feelings of the victim), such as “I can imagine how the victim must feel, even if he or she would not tell.” Responses were provided on a 4-point scale from 0 = *never true* to 3 = *always true*. The total score for empathy was the average of the scores of the seven items (α = .85 at T1; α = .92 at T2).

#### Anti-Bullying Attitudes

Nine items from the Provictim Scale (Rigby & Slee, 1991; for example, “It is wrong to join in bullying” or “Kids who are weak are just asking for trouble”) were rated on a 5-point scale from 0 = *completely disagree* to 4 = *completely agree*. Six of the nine items were recoded so that higher scores reflect stronger anti-bullying attitudes. The total score for anti-bullying attitudes was the average of the scores of the nine items (α = .75 at T1; α = .78 at T2).

#### Peer Status

Within-classroom peer nominations were used to measure perceived popularity and likeability (see Cillessen & Marks, 2011). Perceived popularity was assessed with one item asking participants who they thought was most popular in their class. Likeability was assessed with one item asking participants who they liked the most. For each type of peer status, proportion scores were calculated by dividing the number of received nominations by the number of nominators.

### Analysis Plan

To determine the extent to which students perceived by others as engaging in bullying behavior admit to bullying others or deny it, percentages of peer-reported bullies (i.e., scoring higher or equal to 0.5 *SD* above the same-sex classroom mean) providing each of five response options on the self-report measure of bullying were computed at both time points. The use of these criteria to identify peer-reported bullies was selected to prevent an overrepresentation of boys in the subsample of bullies (Peeters et al., 2010; Solberg & Olweus, 2003), and it is consistent with selection criteria used in other studies utilizing a lenient cut-off (e.g., Juvonen et al., 2003; Schwartz, 2000). As our analyses are conducted with the subsample of bullies, this lenient cut-off was adopted to ensure enough variability within this subsample.

The other study hypotheses were tested in a series of multiple linear regression models, which were run using maximum likelihood estimation with robust standard errors (MLR) and accounting for T1 (or T2 for the last model) within-classroom dependence. The percentage of missing data was low for most variables. There was no missing data for peer-reported bullying, peer-reported victimization, or popularity, and for likeability it was <0.1%. For all other variables, it ranged from 4.7% to 5.1%. These missing data were dealt with using full-information maximum likelihood estimation (FIML). Among the 5,908 students participating at T1, 316 did not participate at T2 due to changing schools or not being present on the day of data collection. Compared with those participating at both time points, those participating at T1 only were more likely to belong to an intervention school (*p* = .048), had higher levels of T1 peer-reported bullying (*p* < .001), victimization (*p* = .003), self-reported bullying (*p* ⩽ .001), and anxiety (*p* = .01), and lower levels of self-esteem (*p* = .01), popularity (*p* = .03), likeability (*p* = .02), and anti-bullying attitudes (*p* = .01).

To test whether bullies’ denial or admission to their behavior was related to specific psychosocial characteristics, we selected a subsample of peer-reported bullies at T1 and examined the effects of T1 victimization, depression, anxiety, self-esteem, perceived popularity, likeability, empathy toward victims, and anti-bullying attitudes on T1 admitting to bullying, controlling for levels of peer-reported bullying, age, and gender. To test whether admitting to bullying others predicts decreases in bullying over time and greater responsiveness to anti-bullying interventions, we again conducted analyses on a subsample of peer-reported bullies at T1. We examined the effects of initial admitting to bullying (T1) on future peer-reported bullying (T2), controlling for T1 peer-reported bullying, KiVa intervention, age, and gender. In a subsequent model, we added an interaction term between KiVa intervention and T1 admitting to bullying, to investigate whether the effects of initial admitting to bullying on future peer-reported bullying differed for students in intervention schools and students in control schools. The two variables included in the interaction term were binary and left uncentered. In these last two models, T1 peer-reported bullying and age were centered at the sample mean. In our last regression model, we examined whether belonging to an intervention school deterred or encouraged admitting to bullying by regressing T2 self-reported bullying on KiVa intervention, controlling for age, gender, and T2 peer-reported bullying, in a subsample of T2 peer-reported bullies.

## Results

### Descriptive Statistics and Correlations

Means, standard deviations, and intercorrelations for all study variables are presented in Table 1. The correlations between peer-reported and self-reported bullying were *r* = .36 at T1 and *r* = .28 at T2, indicating limited overlap between the two variables. At T1, both variables were positively correlated with victimization, depression, and popularity and negatively correlated with self-esteem, likeability, empathy, and anti-bullying attitudes. Only self-reported bullying was significantly associated with anxiety and this association was positive.

**Table 1.** Descriptive Statistics and Bivariate Correlations Among Study Variables.

	*N*	*M* (*SD*)	1.	2.	3.	4.	5.	6.	7.	8.	9.	10.	11.
1. Peer-reported bullying T1	5,908	0.07 (0.12)	–										
2. Self-reported bullying T1	5,769	0.40 (0.70)	.36	–									
3. Victimization T1	5,908	0.06 (0.09)	.22	.14	–								
4. Depression T1	5,629	0.58 (0.60)	.08	.19	.17	–							
5. Anxiety T1	5,609	1.34 (0.86)	.02_a_	.13	.20	.38	–						
6. Self-esteem T1	5,609	2.74 (0.70)	–.11	–.21	–.15	–.55	–.50	–					
7. Popularity T1	5,908	0.16 (0.18)	.15	.04_b_	–.15	–.08	–.13	.08	–				
8. Likeability T1	5,907	0.16 (0.11)	–.12	–.07	–.22	–.08	–.10	.09	.56	–			
9. Empathy T1	5,625	2.03 (0.61)	–.17	–.14	–.01_a_	–.06	.12	.08	.00_a_	.06	–		
10. Anti-bullying attitudes T1	5,628	3.23 (0.63)	–.27	–.25	–.03_c_	–.10	–.08	.24	–.01_a_	.06	.46	-–	
11. Peer-reported bullying T2	5,908	0.06 (0.10)	.76	.32	.16	.07	–.01_a_	–.10	.13	–.07	–.16	–.24	–
12. Self-reported bullying T2	5,579	0.29 (0.62)	.25	.36	.10	.14	.07	–.15	.03_c_	–.04_b_	–.12	–.20	.28

*Note.* All correlations significant at *p* < .001, except for those with the subscripts a (=nonsignificant), b (=significant at .01), and c (=significant at .05); T1 = Time 1 (baseline, end of academic year) and T2 = Time 2 (follow-up, end of subsequent academic year); range of measures: peer-reported bullying, victimization, popularity, likeability = 0−1, self-reported bullying, depression, anxiety, self-esteem, anti-bullying attitudes = 0−4, empathy = 0−3.

To obtain an estimate of how prevalent denial was among bullying perpetrators at each time point, which was our first research goal, we computed, among peer-reported bullies (scoring higher or equal to 0.5 *SD* above the same-sex classroom mean), the proportions of students answering each of five response options on the self-report measure of bullying at both time points (see Table 2). Using this criterion, 22.8% of the T1 participants (*n* = 1,304) and 22.6% (*n* = 1,261) of the T2 participants were identified by their peers as bullying others. The percentage of peer-identified bullies who responded that they had not been bullying at all in the past few months was 48.1% at T1 and 63.3% at T2. These numbers suggest that it is common for peer-reported bullies not to admit to their behavior.

**Table 2.** Percentages of Peer-Reported Bullying Perpetrators Providing Each of Five Response Options on the Self-Report Measure of Bullying.

How often have you bullied another pupil during the last few months?	Time 1 *N* = 1,304	Time 2 *N* = 1,176
Not at all	48.1	63.3
Once or twice	39.2	27.7
2/3 times a month	8.5	5.1
Once a week	2.7	1.6
Several times a week	1.4	2.3

*Note*. At Time 2, there was 6.74% missing data on self-reported bullying. Peer-reported bullying perpetrators score ⩾0.5 *SD* above same-sex classroom mean on peer-reported bullying.

### Regression Analyses

#### Admitting to Bullying and Psychosocial Characteristics

Our second goal was to test whether admitting to bullying was associated with specific psychosocial characteristics for peer-identified bullying perpetrators. In a regression analysis with T1 data only, we examined the effects of eight psychosocial characteristics on admitting to bullying, controlling for peer-reported bullying, among peer-identified bullying perpetrators (Table 3). The model explained 20.7% of the variance in admitting to bullying others. Higher levels of depression and anxiety as well as lower anti-bullying attitudes were associated with a higher likelihood of admitting to bullying. There was no significant effect of victimization, self-esteem, peer status, or empathy on admitting to bullying others. These findings indicate that bullying perpetrators who do not admit to their behavior are less likely to suffer from internalizing problems and tend to score higher in anti-bullying attitudes than those who do admit to their behavior.

**Table 3.** Unstandardized Estimates From Logistic Regression Predicting Admitting to Bullying Among Time 1 Peer-Reported Bullying Perpetrators.

Effect	Estimate	*SE*	Odds ratio	95% CI of odds ratio		*p*
				LL	UL	
Age	–0.012	0.082	0.988	0.842	1.159	.880
Gender (boy)	0.160	0.161	1.174	0.856	1.609	.319
Peer-reported bullying	3.079	0.581	21.744	6.960	67.933	< .001
Peer-reported victimization	1.271	0.717	3.563	0.874	14.530	.076
Depression	0.359	0.135	1.432	1.100	1.865	.008
Anxiety	0.242	0.087	1.274	1.074	1.511	.005
Self-esteem	–0.179	0.117	0.836	0.665	1.052	.126
Popularity	0.570	0.379	1.769	0.841	3.721	.133
Likeability	–0.950	0.759	0.387	0.087	1.712	.211
Empathy for the victim	0.001	0.114	1.001	0.801	1.252	.991
Anti-bullying attitudes	–0.332	0.109	0.718	0.579	0.889	.002

*Note. N* *=* 1,304 (51.9% admit). *R*^2^: .207. CI = confidence interval; LL = lower limit odds ratio; UL = upper limit odds ratio. Time 1 peer-reported bullying perpetrators score ⩾0.5 *SD* above same-sex classroom mean on Time 1 peer-reported bullying.

#### Admitting to Bullying and Changes in Bullying Behavior Over Time

Our third goal was to test whether admitting to bullying others predicted decreases over time in the bullying behavior of peer-identified bullies and their greater responsiveness to the KiVa anti-bullying intervention. Peer-reported bullying at T2 was first regressed on T1 admitting to bullying, controlling for age, gender, T1 peer-reported bullying, and KiVa intervention, among T1 peer-reported bullying perpetrators (see Table 4). The model explained 58.1% of the variance. Admitting to bullying was not significantly associated with peer-reported bullying 1 year later. Therefore, there was no indication that admitting to bullying others was associated with changes in bullying over time. As expected, peer-reported bullying at T2 was lower for students in KiVa schools compared with control schools.

**Table 4.** Predictors (Unstandardized Estimates) of Time 2 Peer-Reported Bullying Among Time 1 Peer-Reported Bullying Perpetrators.

Effect	Estimate	*SE*	95% CI		*p*
			LL	UL	
Main effects					
Intercept	0.121	0.052	0.019	0.223	.020
Age	–0.010	0.005	–0.019	–0.001	.029
Gender (boy)	0.034	0.009	0.016	0.052	<.001
Peer-reported bullying T1	0.633	0.036	0.563	0.704	<.001
Admitting to bullying T1	0.005	0.006	–0.006	0.016	.363
KiVa	–0.023	0.008	–0.038	–0.008	.003
Interactive effects					
Intercept	0.114	0.052	0.012	0.216	.028
Age	–0.010	0.005	–0.019	–0.001	.027
Gender (boy)	0.034	0.009	0.016	0.051	<.001
Peer-reported bullying T1	0.634	0.036	0.563	0.704	<.001
Admitting to bullying T1	0.020	0.008	0.004	0.037	.016
KiVa	–0.009	0.008	–0.025	0.007	.262
KiVa × Admitting to Bullying T1	–0.026	0.012	–0.049	–0.003	.028

*Note. N* = 1,304. For the main effects’ model: *R*^2^: .581. For the interactive effects’ model: *R*^2^: .583. T1 = Time 1; CI = confidence interval; LL = lower limit; UL = upper limit; Time 1 peer-reported bullying perpetrators score ⩾0.5 *SD* above same-sex classroom mean on Time 1 peer-reported bullying.

**Figure 1.**
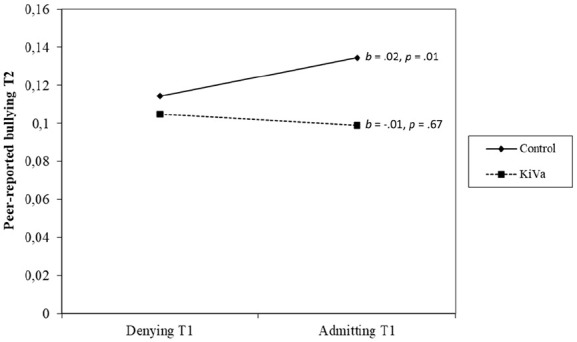
Interaction Between Admitting to Bullying and Participation in the KiVa Program in Predicting Time 2 Peer-Reported Bullying Among Time 1 Peer-Reported Bullying Perpetrators. *N* = 1,304; Both the predictor variable (admitting vs. denying) and the moderator variable (KiVa intervention vs. control) were binary variables; T1 = Time 1 and T2 = Time 2; Time 1 peer-reported bullying perpetrators score ⩾ 0.5 *SD* above same-sex classroom mean on Time 1 peer-reported bullying.

Next, to investigate whether admitting to bullying others would be associated with stronger responsiveness to the KiVa intervention, we added to this model an interaction between KiVa and initial admitting to bullying (Table 4). This interaction, depicted in Figure 1, was significant, indicating that in control schools, those who admitted to bullying at T1 were more likely to continue bullying in T2 than those who denied it (*p* = .013). In KiVa schools, there was an opposite trend, suggesting that those who admitted to bullying at T1 bullied less at T2 than those who denied it at T1. However, the simple slope for KiVa schools was not significant (*p* = .671).

#### Effects of Intervention on Admitting Versus Denying

Our last objective was to test whether participating in the KiVa program deterred admitting to bullying others. Using the subsample of peer-identified bullies at T2, admitting to bullying at T2 was regressed on KiVa intervention (vs. control), controlling for T1 peer-reported bullying, age, and gender (see Table 5). There was no significant effect of KiVa, suggesting that peer-reported bullies are as likely to report bullying others in schools implementing an anti-bullying program as in control schools. Peer-reported bullying at T2 was positively associated with T2 admitting bullying.

**Table 5.** Unstandardized Estimates From Logistic Regression Predicting Admitting to Bullying at Time 2 Among Time 2 Peer-Reported Bullying Perpetrators.

Effect	Estimate	*SE*	OR	95% CI		*p*
				LL	UL	
Age	–0.044	0.081	0.957	0.817	1.120	.582
Gender (boy)	0.208	0.148	1.231	0.921	1.644	.160
T2 peer-reported bullying	2.190	0.489	8.938	3.425	23.326	<.001
KiVa intervention	–0.274	0.142	0.761	0.576	1.005	.054

*Note. N* = 1,261 (36.7% admit); *R*^2^: .058; T2 = Time 2; OR = odds ratio; CI = confidence interval; LL = lower limit; UL = upper limit; Time 2 peer-reported bullying perpetrators score ⩾0.5 *SD* above same-sex classroom mean on Time 2 peer-reported bullying.

## Discussion

Making young bullying perpetrators change their behavior is challenging. Bullying is a relatively stable behavior (e.g., Camodeca et al., 2002; Chu et al., 2018); almost half of childhood bullies have been found to persist into adolescence (Scholte et al., 2007). Although the primary goal of school-based anti-bullying programs is to change the behavior of those who bully their peers and some programs have shown success (Gaffney et al., 2021), some youth continue to bully, even after participating in an effective anti-bullying program (e.g., Garandeau et al., 2014; Hensums et al., 2023). With an eye on better understanding why some students persist in their bullying behavior, this study focused on possible differences between those admitting to bullying others and those denying it among youth identified as bullies by their classmates. First, it examined how prevalent denying bullying was among them (Goal 1). Second, differences between “admitters” and “deniers” were examined for multiple aspects of social and emotional functioning (Goal 2), and changes over time in bullying behavior, including in response to the KiVa anti-bullying program that has been shown to be effective at reducing school bullying (Kärnä et al., 2011; Goal 3). In addition, this study investigated whether participation in an anti-bullying program deterred peer-identified perpetrators from admitting to bullying others (Goal 4).

Regarding the prevalence of denying, among a sample of youth who scored at least 0.5 *SD* above their same-sex classroom mean in peer-reported bullying (20%–22% of participants), almost half responded that they had not bullied at all in the past few months during the initial data collection. This proportion climbed to almost two thirds of peer-identified bullies 1 year later, suggesting that denying is relatively common and may become more common with increasing age. This high prevalence of denying was consistent with past studies showing that the prevalence of bullying tends to be underestimated when based on self-reports (e.g., Cole et al., 2006; Tatum & Grund, 2019). As bullying is an immoral behavior that is against the school rules, it is not surprising that many youth who bully are not willing to admit to it. This can reflect socially desirable responding and therefore be driven by impression management or self-deceptive enhancement (Paulhus, 1988). Some youth may also genuinely feel that their behavior (e.g., making fun of someone) is not bullying. At the same time, it is worth noting that the strongest predictor of admitting among peer-identified bullying perpetrators was the strength of one’s reputation for bullying.

In terms of the correlates of admitting to bullying, findings partly confirmed previous research on the differences between self-reported and peer-reported bullying, such that peer-identified bullies who admitted to having bullied their classmates reported higher levels of depression and anxiety, and were less likely to endorse anti-bullying attitudes compared with those who denied bullying (e.g., Bouman et al., 2012). However, they did not significantly differ in levels of peer status, self-esteem, empathy, or victimization. It is possible that children with preexisting internalizing concerns who bully others may be more willing to admit to engaging in negative behaviors such as bullying. A recent study by Azevedo Da Silva et al. (2020) has shown that internalizing problems led to increases in self-reported bullying perpetration. In this case, bullying behavior may be interpreted as a symptom of other, underlying psychological concerns. If admitters are indeed perpetrators whose behavior is driven by emotional problems, they may be more likely than deniers to engage in overt forms of bullying behavior that is easily noticed by adults. If they have already been confronted by school personnel about their problematic behavior, they may be highly aware of it and not see the need to deny it in a survey. Additional longitudinal research is needed to tease apart the mental health antecedents and consequences of admitters and deniers. Interestingly, deniers reported higher anti-bullying attitudes than admitters. This is consistent with findings of a lower association between moral disengagement (i.e., pro-bullying attitudes) and bullying when bullying is peer-reported than self-reported (e.g., Killer et al., 2019). This could indicate that admitters find bullying justified, possibly due to hostile attribution bias or a more negative perception of peers, which is why they willingly report engaging in the behavior. It might also simply reflect deniers’ tendency for socially desirable responding. Unexpectedly, there were no differences in the peer status of admitters and deniers, indicating that both groups were similar in terms of popularity (higher than average) and likeability (lower than average). This suggests that admitting to bullying others refers to a personal attitude that is either not picked upon by others or has little bearing on how bullying perpetrators are perceived by their peers.

Importantly, contrary to our hypothesis, admitting to bullying was not overall associated with changes in peer-reported bullying behavior over time. Admitting was not the preliminary step toward a positive behavioral change. On the contrary, as suggested by the significant moderating effect of the intervention on the links between admitting and changes in bullying, bullying perpetrators in control schools who admitted to bullying others were more likely to be nominated as bullies by their classmates 1 year later. This might be related to admitters’ lower endorsement of anti-bullying attitudes and higher mental health difficulties. In KiVa schools, there was no significant effect of admitting on later peer-reported bullying, indicating that admitting or denying one’s bullying behavior does not affect students’ responsiveness to an anti-bullying program. Overall, this suggests that admitting to one’s bullying behavior is not a prerequisite for positive behavioral change.

Finally, there was no significant effect of the KiVa program on admitting, indicating that peer-identified bullies are not less likely to report bullying others in schools implementing an anti-bullying program compared with youth in control schools. This finding is encouraging, as it suggests that raising students’ awareness of the seriousness of bullying, as anti-bullying interventions do, does not necessarily lead participants to underreport their bullying behavior.

### Limitations and Future Directions

While this study contributes new information about the implications of admitting to bullying among peer-identified bullies, several limitations deserve to be discussed. First, there are important differences between reporting one’s behavior on an (anonymous) questionnaire and confessing to it when being confronted by adults regarding one’s alleged bullying behavior. In the latter situation, denying one’s actions may be mostly motivated by a desire to avoid disciplinary sanctions. When filling out anonymous questionnaires, social desirability processes are likely to play a role. Therefore, it is unclear whether the current pattern of findings would generalize to face-to-face, targeted interventions that involve direct conversations with youth who have been accused of bullying. Second, it is difficult to determine whether the socially desirable responding that likely explains denying one’s bullying behavior in our study’s anonymous surveys is a process of self-deception (i.e., unconscious, positively inflated personal beliefs about the self) or impression management (i.e., attempting to positively manipulate other’s perceptions of the self). Collecting additional data on emotional and moral mechanisms (e.g., guilt and shame) would be useful in determining whether deniers are consciously aware that they are attempting to deceive others, or whether they do so because they are ashamed to admit their misbehavior to themselves, as well as to others.

Additional limitations are related to the measurement methods used to assess bullying in this study. Peer-reports were used to identify youth who bully others. However, peer-reports are based on reputation, and may be influenced by other sources of information bias. For example, youth who were honest about not bullying others may be nominated by one or a few peers for other reasons (e.g., peer conflict, revenge, as a joke). Thus, in some cases, peer-reports may not reflect actual bullying behavior, and some youth who denied bullying others may have been telling the truth. This may be especially the case given the relatively lenient cut-off used to identify our subsample of peer-nominated bullies. Consequently, the effects observed among deniers should be interpreted with some caution. Perhaps most importantly, there are differences in the measures of self- and peer-reported bullying other than the informant. They are based on different measurement scales, and thus capture distinct constructs—self-report measures assess frequency of behavior, and peer-reports provide an empirical estimation of the strength of one’s reputation among a selected group of individuals. As such, they are not directly comparable. We encourage replication studies of the current findings using measures other than peer-reports to assess actual bullying, such as observations.

Finally, the lower peer-reported bullying scores observed in intervention schools may have been influenced by other factors. It is possible that peer-identified bullies who participated in the intervention reduced their frequency of participation in more direct forms of bullying (e.g., physical, verbal) while continuing to indirectly bully a smaller subset of peers through other means that were not observed by the larger peer group (e.g., social, cyber-bullying). Additional research is needed to determine, first, whether those who admit to bullying others tend to bully in different ways (e.g., more direct, visible ways) compared with those who deny bullying others and, second, whether the implementation of anti-bullying interventions leads to modifications in the type of bullying that perpetrators engage in.

It is also important to consider that the data analyzed for this study were collected about 15 years ago. This may have affected the findings in at least two ways: First, the prevalence of cyberbullying may have been lower back then and the ethnic background of the population is now more diverse. Second, nowadays, the implementation of anti-bullying measures has become more widespread in schools, and the awareness of the issue has increased in the society at large, which could make it even less likely to find differences between intervention and control schools when it comes to admitting to bullying others.

## Conclusion

This study advances the field of school bullying research in several ways: First, it further demonstrates that bullying perpetrators form a heterogeneous group (Peeters et al., 2010), which is important to consider when investigating the psychological processes leading to bullying and determining the best intervention strategies. Indeed, those who admitted to bullying others experienced more internalizing problems and endorsed weaker anti-bullying attitudes than those who denied it. Second, contrary to our expectations, no indication was found that admitting to the behavior in a survey predicted positive behavioral change. Admitting to bullying others, compared with denying it, was actually associated with higher levels of bullying behavior 1 year later in control schools. This could suggest that getting perpetrators to acknowledge their behavior should not be the focus of targeted interventions (i.e., teacher-held discussions with perpetrators in specific cases of bullying), as this acknowledgment may not be key to beneficial outcomes. Rather, having a school context that raises students’ and teachers’ awareness of the problem of bullying may be more important for reducing bullying. Third, the finding that participating in an anti-bullying program did not deter bullying perpetrators from admitting to the behavior is important information for the design of evaluation studies, especially in countries where it is difficult to collect peer-reports.

The next steps in this line of research would be to investigate the effects of admitting to bullying others in face-to-face situations between perpetrators and school personnel. Specifically, future research should examine whether denial impedes the effectiveness of targeted interventions, and if different approaches to tackling bullying are more effective among deniers (see Johander et al., 2022). Deniers may indeed be more receptive to discussions where they are not openly blamed for their behavior. In addition, identifying the direction of effects regarding antecedents and consequences of admitting to bullying would be useful for informing targeted anti-bullying interventions.
